# From early to contemporary normative modeling: Mapping individual differences in neurophysiological signals

**DOI:** 10.1162/IMAG.a.1269

**Published:** 2026-06-15

**Authors:** Francesco Antonio Mallus, Yanwu Yang, Richard Dinga, Mostafa Seyed Kia, Tijl Grootswagers, Abele Michela, Tomas Ros, Thomas Wolfers

**Affiliations:** Department of Psychiatry and Psychotherapy, University Hospital Tübingen, Tübingen, Germany; Graduate Training Center of Neuroscience, International Max-Planck Research School, Tübingen, Germany; Max-Planck Institute for Intelligent Systems, Tübingen, Germany; German Center for Mental Health (DZPG), partner site Tübingen, Germany; Department of Neurology, Jena University Hospital, Jena, Germany; Center of Cognitive Science and Artificial Intelligence, Tilburg University, Tilburg, The Netherlands; Donders Institute for Cognition, Brain and Behavior, Radboud University, Nijmegen, The Netherlands; Department of Psychiatry, UMC Utrecht Brain Center, University Medical Center, Utrecht, The Netherlands; The MARCS Institute for Brain, Behaviour and Development, Western Sydney University, Sydney, Australia; CIBM Center for Biomedical Imaging, University of Geneva, Geneva, Switzerland; Department of Clinical Neuroscience, University of Geneva, Geneva, Switzerland; German Center for Mental Health (DZPG), Jena-Magdeburg-Halle, Jena, Germany; Institute of Psychology, Friedrich Schiller University Jena, Jena, Germany

**Keywords:** normative modeling, electroencephalography (EEG), magnetoencephalography (MEG), psychiatric diagnostics, psychiatric disorders, mental disorders, neurological diseases

## Abstract

Normative modeling has become a cornerstone of computational neuroscience, offering a powerful framework for detecting individual deviations from typical brain function. This review traces its trajectory in electrophysiology of the brain, from early studies in the 1970s, through a period of relative neglect, to its recent revival driven by machine learning advances and the availability of large-scale datasets. We provide a structured overview of this evolution, showing the shift from small, site-specific age-based models to increasingly harmonized approaches that integrate diverse biological and methodological innovations. Key studies are compared with respect to cohort composition, modeling strategies, and validation procedures, situating each within the broader arc of methodological progress. Despite this momentum, significant challenges remain, such as a lack of standardized practices, limited comparability across studies, and the need for richer integration of complex neurophysiological signals. Looking ahead, we argue that the future of electrophysiological normative modeling lies in scaling and unifying efforts, through international collaborations, standardized pipelines, and the incorporation of novel features. By coupling machine learning with both cross-sectional and longitudinal designs, the field can progress from proof-of-concept demonstrations to precise, individualized brain mapping. Ultimately, such advances will provide the foundation for clinical applications, enabling cost-effective personalized treatment monitoring and a more refined understanding of individual differences in complex brain disorders.

## 1 Introduction

In recent years, the convergence of advanced computational methods (Badrulhisham et al., 2024; Islam et al., 2019; Király & Hangya, 2022; Kriegeskorte & Douglas, 2018; Liang et al., 2022), cutting-edge neuroscientific techniques (Jadhav et al., 2022; Ranasinghe & Mapa, 2024; Sejnowski et al., 2014), and the growing emphasis on personalized medicine (Keizer, 2021; Mathur & Sutton, 2017) has opened up opportunities to transform diagnosis, treatment, and monitoring of neurological and mental disorders. These conditions are highly prevalent and affect nearly every family (World Health Organization, 2021), and traditional diagnostic approaches often struggle to capture their complexity and heterogeneity (Neves et al., 2024; Rahul et al., 2024; Winter et al., 2022; Zabihi et al., 2020). Normative modeling has emerged as an analytical framework to address this limitation by constructing population-based reference models or “norms” from large-scale datasets involving thousands of individuals (A. F. Marquand et al., 2019; Rutherford et al., 2023; Wolfers et al., 2021). These models enable clinicians and researchers to detect statistically meaningful deviations from various biomarkers at the individual level. When applied to electrophysiological data, such as electroencephalography (EEG) and magnetoencephalography (MEG), normative modeling permits the characterization of individual-level brain dynamics across multiple dimensions, including, but not limited to, spectral power, connectivity, and temporal dynamics (Biasiucci et al., 2019; Binnie & Prior, 1994; Hari & Salmelin, 2012). This multidimensional charting facilitates the identification of typical and atypical neural patterns, yielding a comprehensive map of neural functioning (Buzsáki & Draguhn, 2004; Jeste et al., 2015; Keizer, 2021; Nuwer, 1997). Such mapping is fundamental for personalized medicine, enabling interventions tailored to individual patients, improving treatment monitoring, refining diagnostic precision, and providing cost-effective quantification of brain function at the level of the individual (John et al., 1977; Thatcher et al., 2003).

To contextualize the following literature review, we briefly trace the evolution of normative modeling for neurophysiological signals, from its foundational studies (John et al., 1977; Thatcher, 1998; Thatcher et al., 2003) to recent methodological and conceptual advances (Kia et al., 2021; Rutherford et al., 2023; Wolfers et al., 2021). Early models primarily focused on defining norms using simple parametric approaches (Bosch-Bayard et al., 2020; John et al., 1980; Matoušek & Petersén, 1973; Valdés et al., 1990), often applied to small samples, unrepresentative of the broader population. In contrast, contemporary normative modeling leverages large-scale datasets (Bozek et al., 2023; Brady et al., 2020; ENIGMA Clinical High Risk for Psychosis Working Group et al., 2024; Little et al., 2024; Winter et al., 2022) and modern machine learning frameworks to enhance precision and robustness of learned trajectories (Bozek et al., 2023; C. J. Fraza et al., 2021; Ge et al., 2024). These advanced approaches integrate high-dimensional data (Kaplan et al., 2014; Zabihi et al., 2021), nonlinear modeling (Ko et al., 2021; Young et al., 2018), and uncertainty quantification (Kia & Marquand, 2018a, 2018b), making them more robust for applications in psychiatry and neurology (Rutherford et al., 2023; Kia et al., 2021; A. Marquand et al., 2024; Wolfers et al., 2020). Notably, while early studies predominantly relied on EEG, contemporary normative modeling is rooted in imaging methodologies such as magnetic resonance imaging (MRI) (Bethlehem et al., 2020; Wolfers et al., 2018; Zabihi et al., 2020) and functional MRI (fMRI) (Parkes et al., 2020; Savage et al., 2024). In general, MRI readouts provide higher spatial but much lower temporal resolution than EEG or MEG, which additionally measure neuronal activity more directly (Liljeström et al., 2009; Sharon et al., 2007) as potential differences in electrical activity or their associated magnetic field, respectively.

In this review, we provide a comprehensive overview of normative modeling approaches applied to EEG and MEG. In contrast to the neuroimaging literature, where normative modeling has been described in detail (Alyas et al., 2025; C. Fraza et al., 2025; Maccioni et al., 2026), previous reviews on electrophysiology of the brain are outdated (Thatcher & Lubar, 2009). They fall short in providing a comprehensive overview and lack a definition of contemporary normative modeling (Keizer, 2021). This review highlights EEG and MEG as unique methodologies that enable cost-effective normative frameworks while posing challenges that require tailored methodological solutions. Clarifying the evolution and current state of normative modeling in electrophysiology of the brain is essential to understanding how the renewed interest in this approach can drive advances in brain signal analysis, analogous to recent progress in neuroimaging (Eickhoff et al., 2016; Kim et al., 2023; Tardif et al., 2016). For this reason, the overarching goal of this review is to trace the early foundations, outline current methodologies, and explore future directions in normative modeling of neurophysiological signals of the brain, anticipating its comeback rooted in increasingly larger samples and advanced machine learning methodologies.

## 2 What is Normative Modeling?

**Fig. 1.**
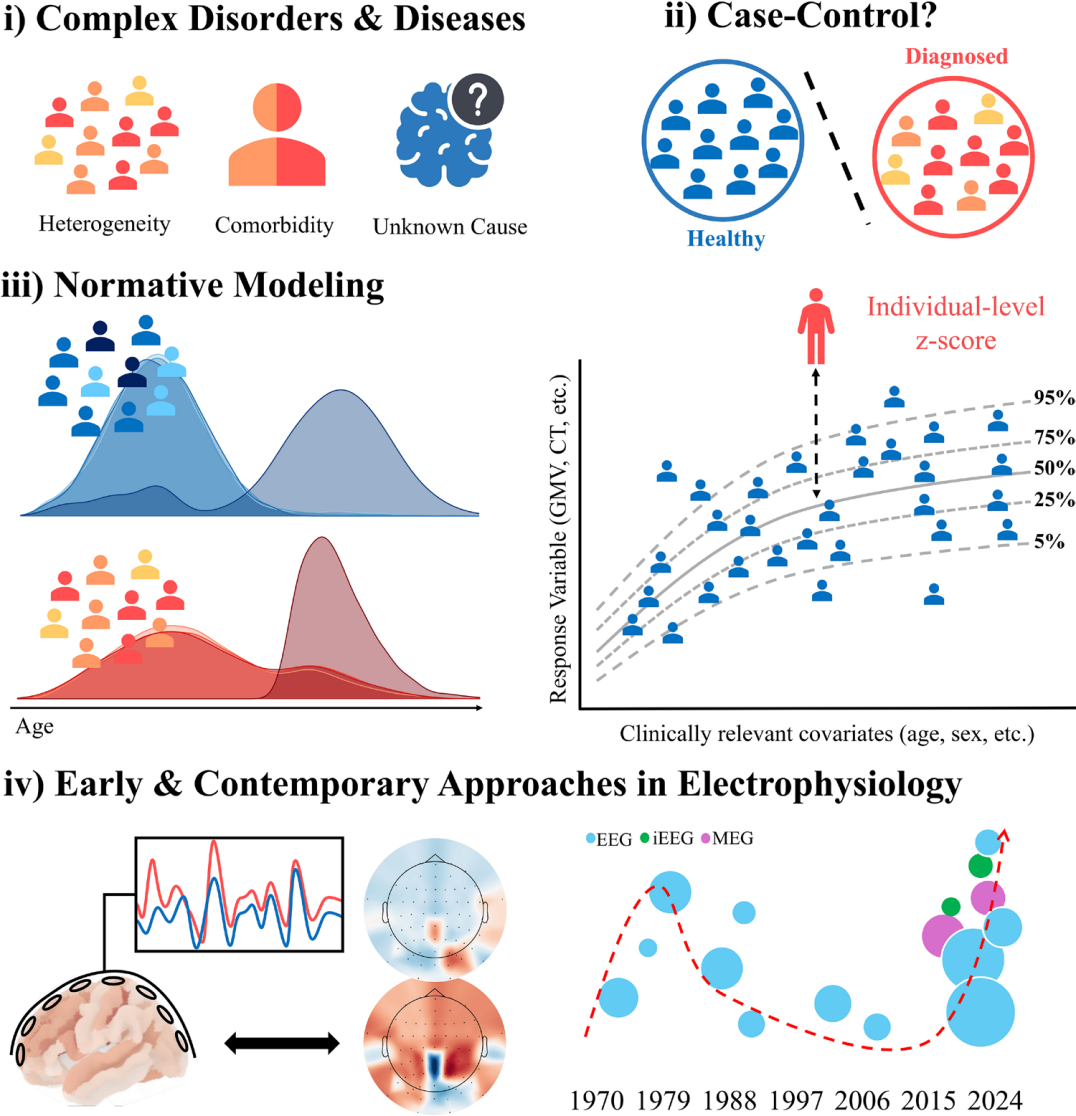
Normative modeling from motivation to application. (i) Complex disorders and diseases are characterized by pronounced heterogeneity and frequent comorbidity, with underlying causes that are often uncertain and differ across individuals. (ii) Classical case–control designs impose a symmetry between “patient” and “control” groups by assuming well-defined and discrete class boundaries. This framing masks the continuous nature of neurobiological variation. Individual differences that span or cut across diagnostic categories are often averaged out or misattributed. (iii) Normative modeling directly addresses this limitation by replacing group contrasts with deviation-based representations, in which each individual is characterized relative to a conditional population-level reference distribution rather than a binary class boundary. Conceptually analogous to growth charts, normative models map individual profiles onto age-, sex-, or context-conditioned reference trajectories, thereby breaking the group-average symmetry and enabling disease modeling at the level of the individual while capturing variation across the full reference population. (iv) Early applications of normative concepts to electrophysiological brain measurements in the 1970s were constrained by small samples, coarse features, simple algorithms, and limited computational resources. In contrast, contemporary normative modeling, enabled by modern machine learning and large-scale cohorts, provides a scalable and cost-effective framework for deriving clinically meaningful markers from electrophysiological measures such as EEG and MEG. This development complements magnetic resonance imaging-based normative models and expands opportunities for scalable and cost-effective quantification of individual deviations in complex brain disorders.

Normative modeling is an analytical framework that quantifies how individuals deviate from typical patterns observed in a reference population (Fig. 1). Inspired by early applications in pediatrics, such as growth charts developed in the early 20th century, normative models provide a statistical reference against which individual measurements can be evaluated (Cole, 2003, 2012; Dewey et al., 1995; Grummer-Strawn et al., 2010; Largo et al., 1978; Wei et al., 2023). These principles were formalized into diagnostic tools by the National Center for Health Statistics in 1977 (Cole, 2012; Grummer-Strawn et al., 2010) and later adopted globally by the World Health Organization (Dewey et al., 1995; Weltgesundheitsorganisation, 2006; World Health Organization, 2024), where they remain widely used to monitor development and identify early signs of atypical maturation (“A Health Professional’s Guide to Using Growth Charts,” 2004; Milani et al., 2012). Over time, both the concept and methodological implementation of normative modeling have evolved substantially. Conceptually, normative modeling shifts the analytical focus from identifying average differences between groups to quantifying how individuals vary relative to population expectations (A. F. Marquand et al., 2016; Wolfers et al., 2020; Wu & Lin, 2023; Zabihi et al., 2020).

In this framework (Su et al., 2024; Wu & Lin, 2023), biological measurements are modeled as covariate-conditioned distributions estimated from a reference population. Individual observations can then be expressed as deviations from these expected values. Importantly, normative modeling does not assume that deviations correspond to predefined diagnostic categories but instead allows biological variation to be examined independently of existing nosological boundaries. Representing individuals in such a reference space across multiple biological features enables visualization of within-group heterogeneity and between-group separation without imposing a priori assumptions about class membership. The resulting multidimensional deviation profiles can be interrogated using both univariate and multivariate analytical approaches. Because deviation scores are derived from feature-wise models that explicitly adjust for relevant covariates, they retain a direct correspondence with the underlying biological measurements. Together, these deviations define an individual’s position relative to the normative reference space.

Such deviation profiles can be shared across individuals with similar biological characteristics and may therefore corroborate existing diagnostic groupings (Tabbal et al., 2025; Wu & Lin, 2023). Alternatively, they may uncover structured heterogeneity within broad diagnostic categories (Ebadi et al., 2025; Woodhouse et al., 2025), motivating finer-grained subtyping or a redefinition of diagnostic boundaries based on quantitative biological markers rather than solely on behavioral or qualitative assessments. This approach contrasts with traditional group-based comparisons, which often overlook within-group variability and comorbidity, particularly in heterogeneous conditions such as neurodegenerative diseases (Levenson et al., 2014; X.-L. Li et al., 2014), neurodevelopmental disorders (Hesdorffer, 2016; Kessler & Merikangas, 2004; A. F. Marquand et al., 2016), and mental disorders (Kessler & Merikangas, 2004; McGrath et al., 2020).

Importantly, deviation profiles are not intended to provide diagnostic classifications but instead quantify biological variation relative to population-level expectations, which may or may not correspond to established clinical entities. Extreme deviations in specific biological measures can highlight potentially relevant processes, but they must be interpreted cautiously and in conjunction with comprehensive clinical evaluation. Normative modeling should, therefore, not be viewed as a stand-alone diagnostic tool. Rather, it provides a principled framework for identifying biological patterns that are more or less typical within a population and for quantifying how individuals deviate from these expectations. Such deviations can help uncover underlying mechanisms that may remain hidden when heterogeneous individuals are grouped under the same diagnostic label despite substantial biological differences. For example, two individuals may present with similar symptoms but for fundamentally different reasons, such as chronic social isolation in one case and a neurobiological imbalance in another. While the observable symptoms may appear comparable, the underlying causes, and consequently their deviation profiles across relevant biomarkers, may differ markedly. Normative modeling can make such differences visible, whereas traditional group-based analyses risk obscuring them when these individuals are analyzed within the same diagnostic category. A key research priority is, therefore, the construction of more representative reference populations (Rutherford et al., 2024), as the interpretability and generalizability of deviation scores depend strongly on the population from which the normative distribution is estimated (Genin et al., 2024).

Normative modeling is largely agnostic to the origin and nature of input features. Anatomical measures such as cortical thickness, subcortical volumes, or white matter integrity capture the structural substrate of brain organization across the lifespan (Berthet et al., 2024; Kim et al., 2023, 2025; Rutherford, Fraza, et al., 2022; Wolfers et al., 2018). Functional measures, including spectral power, connectivity patterns, or network dynamics derived from techniques such as EEG and MEG, reflect ongoing neural activity and are particularly sensitive to state-dependent processes and rapid pathological changes (M. Li et al., 2022; Otero et al., 2011; Rempe et al., 2023; Wu & Lin, 2023; Zamanzadeh et al., 2026). While each feature class provides complementary insights into brain health, neither anatomical nor functional measures alone can fully capture an individual’s neurobiological state. Integrating multimodal features within a unified reference space allows deviations to be quantified simultaneously across structural and functional dimensions (Fotiadis et al., 2024; Kumar et al., 2023; J. R. Taylor et al., 2017; Zhang et al., 2024). Such approaches also enable practical implementations in which independently trained normative models can be combined without requiring access to the original primary datasets, provided that the underlying reference cohorts are representative.

In neuroscience, early attempts at normative modeling date back to the 1960s when NASA explored brain function in astronauts (Adey & Proctor, 1965). One of the first systematic approaches to modeling neurodevelopmental trajectories is attributed to Matoušek and Petersén (1973), who used age-dependent EEG spectral features and representative cohorts to define developmental norms. Subsequent work by Thatcher et al. (Thatcher & Lubar, 2009; Thatcher, 1998), and John et al. (1977, 1980) expanded these ideas and developed pediatric and lifespan EEG growth curves. These early models, however, were limited by relatively small cohorts that often consisted of highly selected “super-healthy” individuals (Kaplan et al., 2014; A. F. Marquand et al., 2016; Wolfers et al., 2018). They typically focused on classical EEG features such as spectral power in canonical frequency bands (John et al., 1980; Thatcher & Lubar, 2009; Thatcher et al., 2003; Valdés et al., 1990) and relied on linear regression approaches that struggled to capture the nonlinear and heterogeneous nature of neurophysiological signals (Benkeser et al., 2018; F. K. Ho & Cole, 2023). Furthermore, most early models ignored heteroscedasticity and non-Gaussian distributions of biological measures, potentially leading to biased estimates of normative ranges (Aswani et al., 2011; Cattaneo et al., 2015; Dinga et al., 2021). These limitations have motivated a methodological shift toward more flexible and data-driven approaches capable of capturing the full reference distribution.

*Contemporary normative modeling* has emerged alongside the availability of large-scale datasets, advances in machine learning, and the growing emphasis on personalized medicine (ENIGMA Clinical High Risk for Psychosis Working Group et al., 2024; Keizer, 2021; Mathur & Sutton, 2017) and standardized clinical frameworks (N. J. Brown, 2012). In this review, we define contemporary normative modeling by three essential components. First, a carefully selected reference cohort must capture the relevant demographic and biological variability of the population of interest (A. Marquand et al., 2024; A. F. Marquand et al., 2016; Rutherford, Fraza, et al., 2022). The composition of the reference cohort is critical, as individual-level deviations are defined relative to this estimated population distribution. Poorly chosen reference data can introduce systematic bias and limit generalizability across age ranges, cultural backgrounds, or clinical populations. Ideally, the reference cohort should resemble the population to which the model will ultimately be applied. Second, normative modeling requires algorithms capable of estimating the conditional distribution of biological measures, including both central tendency and variability, as a function of relevant covariates (De Boer et al., 2024; Kia et al., 2020; Tabbal et al., 2025; Zabihi et al., 2025). Unlike classical predictive models that focus on estimating a single expected value, normative models aim to characterize the full range of typical variation within a population. This enables the computation of individual-level deviation scores, such as z-scores or centiles. Accurate estimation of these ranges requires methods that explicitly account for heteroscedasticity, where variability changes as a function of covariates. Contemporary approaches, therefore, often employ flexible probabilistic models or non-parametric methods to capture complex relationships between covariates and biological measures. Third, rigorous out-of-sample validation is essential to ensure that deviation scores generalize to new datasets and reliably quantify individual differences relative to the estimated norms. This is particularly important in multi-site settings, where differences in acquisition protocols, hardware, or preprocessing pipelines can introduce systematic variability. Effective validation, therefore, includes evaluating model calibration, assessing error distributions, and verifying that observed deviations correspond to meaningful biological or clinical phenomena in independent cohorts (Kia et al., 2020; Kim et al., 2023; Wolfers et al., 2018; Zabihi et al., 2020). Ideally, validated deviation scores should show interpretable relationships with relevant phenotypes such as cognitive performance, symptom severity, or disease progression.

In summary, normative modeling characterizes individual biological variation relative to population expectations (A. Marquand et al., 2024; A. F. Marquand et al., 2016; Rutherford, Kia, et al., 2022). By constructing a statistical reference space from quantitative biological measures, such as anatomical, functional, or molecular features, individuals can be positioned within this space and interpreted in terms of typical or atypical patterns. This framework enables biologically informed stratification and provides a principled basis for studying heterogeneity across individuals, populations, and disease processes.

## 3 Measuring Electrophysiological Signals of the Brain

EEG and MEG provide a window into neural activity by capturing the electrical and magnetic signals generated by large populations of neurons (Biasiucci et al., 2019; Thatcher & Lubar, 2009). Both techniques offer millisecond-level temporal resolution (Rangayyan, 2015; Sharon et al., 2007), enabling the study of rapidly evolving electrophysiological correlates of cognitive and pathological processes (Brookes et al., 2022; Ranasinghe & Mapa, 2024; Stancin et al., 2021; Young et al., 2018). Although EEG and MEG share many advantages (Sharon et al., 2007; Wendel et al., 2009), they measure distinct physical aspects of neural activity and, therefore, provide complementary information about brain function (Biasiucci et al., 2019; Chowdhury et al., 2015; Cohen & Cuffin, 1983; Huizenga et al., 2001; Sharon et al., 2007).

EEG is the most widely used non-invasive modality for monitoring real-time brain activity (Jadhav et al., 2022; Loo & Makeig, 2012), with scalp EEG representing the predominant approach in clinical settings (Jeste et al., 2015; Hegazy & Gavvala, 2022; Quigley, 2022). EEG measures changes in electrical potential generated by neuronal populations, capturing signals originating from both tangential sources in the cortical sulci and radial sources in the gyri (Baillet, 2017; Hämäläinen et al., 1993). Its portability, relative affordability, and ease of deployment make EEG a versatile tool for research and clinical applications (Nuwer, 1997; Rahul et al., 2024). However, the spatial precision of EEG is limited by signal attenuation and volume conduction as electrical currents propagate through the brain, skull, and scalp tissues (Biasiucci et al., 2019).

EEG signals are commonly processed to extract quantitative features that summarize relevant properties of the recorded time series, an approach often referred to as quantitative EEG (qEEG) (Edwards, 2019; John et al., 1977; Keizer, 2021; Livint Popa et al., 2020; Petsche, 1970; Szava et al., 1994). qEEG encompasses a broad set of features that characterize neural activity, including measures of spectral power, connectivity, and temporal dynamics (Biasiucci et al., 2019; Jobst et al., 2020; Nuwer, 1997). Representing electrophysiological data through such feature sets facilitates large-scale analyses and improves interoperability across datasets. This feature-based representation helps overcome common data-sharing constraints across recording sites and mitigates compatibility issues arising from differences in acquisition parameters and experimental setups (Bringas-Vega et al., 2022; Coburn et al., 2006; Prichep, 2005). As a result, qEEG features can be standardized and harmonized across acquisition centers, making them well suited for integration into large multi-site datasets (Coburn et al., 2006; Ko et al., 2021; M. Li et al., 2022; Thatcher & Lubar, 2009).

Intracranial EEG (iEEG), also known as electrocorticography, provides recordings directly from the cortical surface or from depth electrodes implanted in brain tissue, offering substantially higher spatial resolution than scalp EEG (Cooper et al., 2014; Q. Yin et al., 2023). Although iEEG is invasive and, therefore, primarily used in specific clinical contexts such as epilepsy surgery planning (Jobst et al., 2020; Khuvis et al., 2021), recent studies have explored its potential for constructing reference curves of electrophysiological activity. Such efforts may provide valuable insights into normative brain dynamics, albeit derived from recordings obtained in clinical populations rather than from healthy individuals (Frauscher et al., 2018; Jobst et al., 2020).

MEG measures the magnetic fields generated by synchronized neuronal currents, primarily arising from pyramidal neurons oriented tangentially to the scalp, commonly located within sulci of the cortex. Because magnetic fields are less distorted by the surrounding tissues than electrical potentials, MEG signals are less susceptible to volume conduction and signal attenuation than EEG recordings (Van Den Broek et al., 1998). This property can improve spatial localization of neural activity. MEG acquisition also involves a streamlined setup that avoids time-consuming electrode placement. However, MEG systems are typically expensive, non-portable, and technically demanding to operate (Cohen, 1972; Hegazy & Gavvala, 2022; Tiège et al., 2017), particularly when compared with EEG instrumentation (Bhavnani et al., 2022). Although recent technological advances, including new sensor technologies and wearable MEG systems, have begun to mitigate some of these limitations (Brickwedde et al., 2024; Brookes et al., 2022; Pedersen et al., 2022), MEG remains primarily confined to specialized research and clinical environments, most commonly in the context of pre-surgical evaluation (Brickwedde et al., 2024; Hegazy & Gavvala, 2022; Tiège et al., 2017).

When applying normative modeling to electrophysiological data, it is important to recognize that no sharp decision boundary is likely to exist that perfectly separates individuals with mental disorders from healthy individuals based solely on EEG or MEG signals. Electrophysiological features should, therefore, not be interpreted as direct diagnostic markers. Instead, they are best viewed as components of a multidimensional biological profile that captures graded variation across individuals. Within this framework, normative models provide a principled way to characterize such variation by quantifying how individual electrophysiological brain features deviate from population expectations. This approach enables the systematic investigation of heterogeneity both within and across diagnostic categories, supporting data-driven stratification without presupposing discrete disease boundaries. Consequently, when applied to EEG and MEG data, normative modeling is not intended to perform binary classification. Rather, it enables individualized characterization, where deviation scores can support clinical interpretation, risk stratification, and hypothesis generation about potential disease mechanisms. In this way, the analytical focus shifts away from traditional diagnostic categories, which often map only loosely onto underlying biology, toward a more biologically grounded description of individual variation (T. Insel, 2013; T. R. Insel, 2009, 2014).

## 4 Review Approach and Selection Criteria

This work constitutes an in-depth narrative review and conceptual analysis of previously published literature. All referenced papers were carefully scrutinized and evaluated with respect to their scientific content, methodology, and conclusions. No new data were collected, processed, or analyzed as part of this study. Consequently, formal ethical approval and informed consent were not required. All sources are appropriately cited to acknowledge the original authors’ contributions and to ensure transparency and academic integrity.

The focus of this review was shaped by a set of targeted keywords aimed at identifying the most relevant literature (Supplementary Fig. S1). Our iterative search spanned multiple scientific databases and journal platforms, including PubMed, Google Scholar, ScienceDirect, Frontiers, and the Journal of Neuroscience. To ensure broad coverage and capture the historical evolution of the field, we did not apply any restrictions on publication date. The search strategy combined keywords related to normative modeling (“normative model”, “normative database”), signals, and target task (e.g., “EEG”, “resting state”). Initial automated filters were applied to prioritize full-text availability (open access or university access) and to focus solely on research articles.

**Fig. 2.**
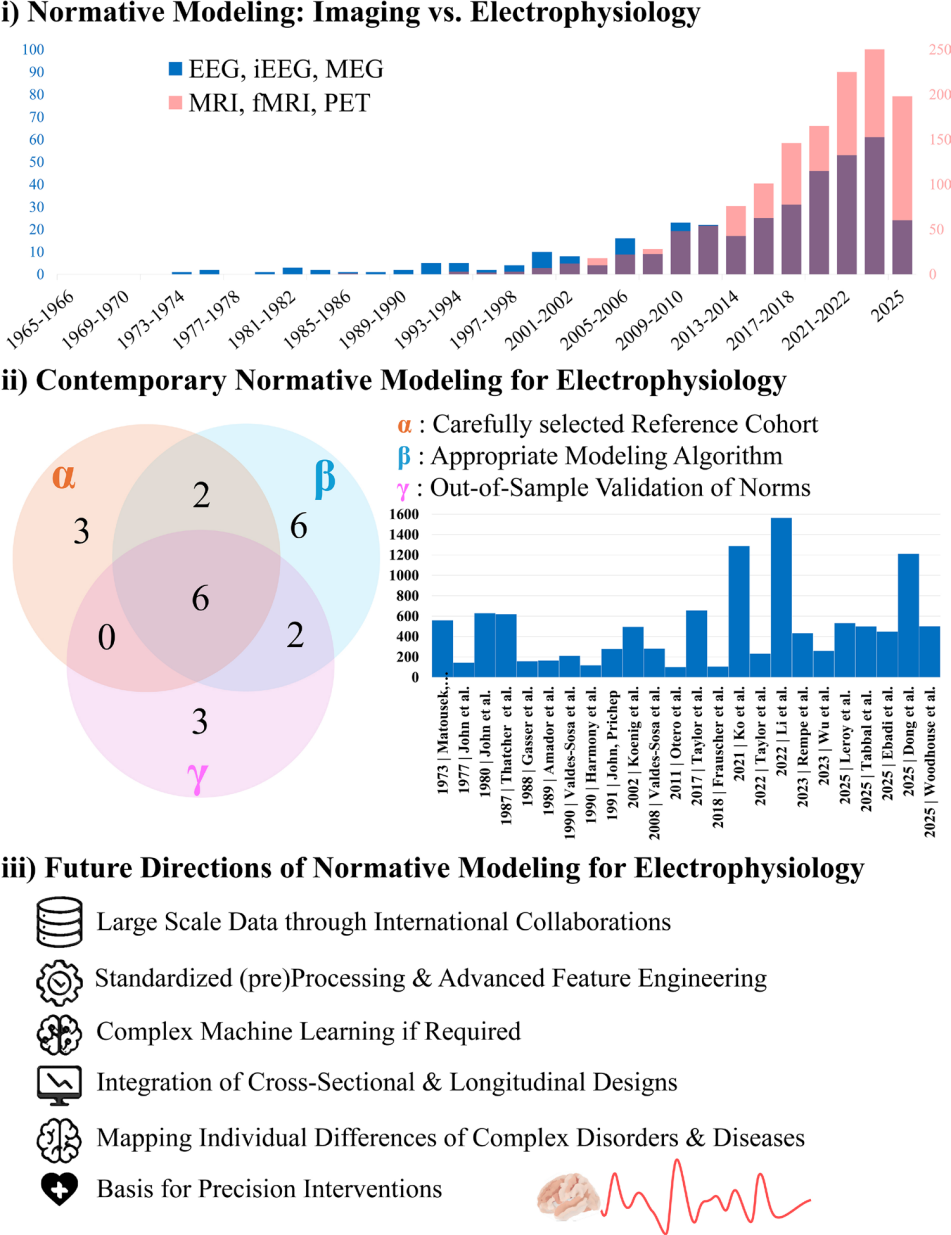
Contemporary normative modeling of electrophysiological brain signals. (i) Early normative modeling was introduced into electrophysiological research as early as the 1970s, well before its adoption in neuroimaging. However, these early studies rarely adhered to the methodological standards that now define contemporary normative modeling, particularly as developed in imaging. As a result, findings were fragmented, difficult to reproduce, and of limited translational value. (ii) We systematically evaluated the electrophysiological normative modeling literature against contemporary criteria. A Venn diagram illustrates the extent to which studies meet the three core requirements of contemporary normative modeling: α, use of carefully selected and representative reference cohorts; β, application of appropriate and flexible modeling algorithms; γ, rigorous out-of-sample validation of individual deviation scores. Our analysis shows that most early EEG and MEG studies meet none or at most one or two of these requirements. This indicates that the true potential of normative modeling in electrophysiology remains unrealized. (iii) We propose that meaningful progress will come from scaling and standardization. Specifically, we foresee large-scale international collaborations to expand sample sizes, harmonized processing pipelines to reduce methodological variability, integration of complex machine learning approaches, and the fusion of cross-sectional with longitudinal designs. Such developments will allow normative modeling in electrophysiology to move beyond proof-of-concept, toward mapping individual differences with high fidelity. Ultimately, this will provide a robust basis for cost-effective precision interventions.

Our preliminary search across PubMed (n = 713), Google Scholar (n = 630), and ScienceDirect (n = 811) yielded a total of 2,154 records (Fig. 2i). After removing duplicates and applying automated filters, 180 unique records remained and were screened based on their titles and abstracts. All records then underwent a rigorous full-text assessment to determine their eligibility for inclusion. Next, we set specific and stringent inclusion criteria designed to identify studies central to EEG- and MEG-based normative modeling. First, eligible studies had to explicitly present regression models, with conditionals, and estimates of population-level variability. Second, only studies using EEG (or iEEG) and MEG signals recorded during resting-state conditions (eyes open, eyes closed, or both) were considered. Studies focused solely on task-based paradigms or sleep-related models were excluded. We identified 25 unique publications for in-depth analysis and summary in Table 1 (visual representation in Supplementary Fig. S2). For each publication, we extracted a comprehensive set of characteristics, including the year of publication, reference cohort, signal type, analytical pipeline, modeling approach, strategy for model construction, and the principal findings or reported outcomes (Supplementary Material).

**Table 1.** Neurophysiological signals normative modeling studies.

								Contemporary normative model		
Authors (year)	Reference cohort (ages)	Signal type	Features	Covariates	Modeling algorithm	Validation cohort (#, ages)	Results	i	ii	iii
Matoušek & Petersén (1973)	560 HC (1–21)	raw EEG	aPSD, rPSD, power ratios	age	Multivariate statistical analysis, fourth-order interpolation	Verified expansive process (46, 14–74)	Age-dependent quotient had the highest correlation with the visual evaluation outcome	No	No	No
John et al. (1977)	144 HC (~20–35)	raw EEG	aPSD, rPSD, AER	age	Age-wise z-scoring stepwise discriminant analysis, numerical taxonomy (clustering)	Various neurological diseases (177)	Classified neurological disease with up to 87% accuracy (vs. conventional EEG). Cognitive impairment in elderly detected with 91–96% accuracy.	No	No	No
John et al. (1980)	630 HC (6–16)	raw EEG	rPSD	Age, sex, site, socioeconomic class, ethnicity	Chebyshev polynomial regression	HC (140, 5–21) HC (Alvarez et al., 1987) (97, 7–11)	Regression coefficients could be independent of cultural, ethnic, socioeconomic, sex, or age factors	No	Yes	Yes
Thatcher et al. (1987)	620 HC (0.2–82)	qEEG	PSD, coherence, amplitude asymmetry	age	Age-group measures standardization & z-scoring	Cross-validation (k-fold)	Computed z-scoring followed a Gaussian distribution, ensuring accurate standardization for EEG feature comparisons	Yes	No	No
Gasser et al. (1988)	158 HC (6–17)	qEEG	PSD	Age, sex	Polynomial regression	n/a	No sex differences and no pubertal spurt, topological effects in power and correlation	No	Yes	No
Amador et al. (1989)	165 HC (5–12)	Raw EEG	PSD, ξα -model parameters	Age	Linear regression, z-scoring	n/a	Systematic local antero-posterior gradient differences in the ξα parameters. The first 4 factors of ξα models correlate to band powers	No	Yes	No
Valdés et al. (1990); Bosch-Bayard et al. (2020); Szava et al. (1989); Valdés et al. (1992)	211 HC (5–80)	qEEG	Narrow band log-spectra	Age, sex	Regression models, z-scoring	HC (129, 5–11), Childhood malnutrition (129, 5–11)	Age-dependent variations: α peak frequency increased from childhood to adulthood and decreased in older age	Yes	Yes	Yes
Harmony et al. (1990)	118 HC (6.4–12.9)	qEEG	aPSD, rPSD	Age, sex, SES, biological risk factors	Linear regression, z-scoring	n/a	Male children showed higher alpha and lower delta band power. Higher frontal lobes aPSD and higher relative delta power in occipital and temporal lobes in low SES individuals	No	Yes	No
John & Prichep (1991)	76 HC + 203 Cognitive impairment (6–88)	EEG	PSD, ERP	Age, Global Deterioration Scale (GDS)	Age-regressed z-scoring, stepwise discriminant function analysis	Cross-validation, HC (60), depression (34), alcoholism (40), dementia (62), Longitudinal (39)	Distinguished HC from individuals with depression, alcoholism, and dementia with 78% accuracy. Predicted future cognitive decline in mildly impaired individuals (GDS=2) over 3–5 years.	No	No	Yes
Koenig et al. (2002)	496 HC (6–80)	Raw EEG	Four classes of microstate, occurrence, duration	Age	Age-wise statistical analysis (mean, std), ANOVA	n/a	Duration and occurrence of microstates were found to change systematically with age, reflecting the maturation of brain functional organization.	Yes	No	No
Valdés Sosa (2008), Valdes-Sosa et al. (2021)	282 HC (18–68)	qEEG, MRI	log(PSD), structural brain metrics	Age, sex, education, handedness	Regression models, Z-score generation from normative data	ADHD (15, 7–18)	Significant deviations in QEEG patterns between the ADHD group and the normative population	Yes	Yes	No
Otero et al. (2011), West et al. (2006)	101 HC (0–1)	qEEG	Broad-band PSD	Age (months)	Polynomial regression, z-scoring	50 HC 1 severe perinatal asphyxia, 1 epileptic seizure	Linear and quadratic age effects. Delta power decrease. Theta, alpha, and beta relative powers and mean frequency increase with age	No	Yes	Yes
J. R. Taylor et al. (2017)	656 HC (18–87)	MEG, MRI, fMRI	Functional connectivity, structural brain metrics	Age, sex, education level, socioeconomic status	Multivariate analysis, univariate GLM	mTBI (25, 20–59) (Itälinna et al., 2023)	For the support vector machine classifier (mTBI vs HC), the most discriminative features were θ-band (4–8 Hz) deviations of the parietal cortex.	Yes	Yes	Yes
Frauscher et al. (2018)	Non-epileptic areas of epilepsy patients, 106 (11–54)	iEEG, MRI	Band-wise relative power peaks	Brain region	Spectral clustering and statistical comparison	Drug-resistant epilepsy (60, 3–29)	Connectivity abnormalities are more effective than univariate neural activity in identifying seizure onset zones. Longer epilepsy diagnoses correlate with greater connectivity abnormalities.	No	No	Yes
Ko et al. (2021)	1289 HC (4.5–81)	Raw EEG	log(PSD)	Age, sex	GAM	HC (244, 5–90), qEEG-Pro^51^, 1 ADHD (10), 1 aMCI (78), 1 Anxiety (43)	Significant variations in EEG patterns between males and females, as well as across different age groups	Yes	Yes	Yes
P. N. Taylor et al. (2022)	Non-epileptic areas of epilepsy patients, 234 (n/a)	iEEG, MRI	rPSD	Brain region	Spectral clustering and statistical comparison	UCLH Epilepsy Cohort (62, 11–54)	Band power variations followed healthy patterns, while abnormalities in spared regions distinguished persistent seizures and patient outcomes	No	Yes	Yes
M. Li et al. (2022)	1564 HC (1–97)	qEEG	Harmonized log- cross-spectral tensors	Age, site, acquisition device	Additive mixed-effects kernel regression, Stable Sparse Robust Classifier (SSRC)	Early life malnutrition (62), COVID-19-related brain dysfunction (237)	The harmonized age- and site-adjusted norms significantly enhanced diagnostic accuracy	Yes	Yes	Yes
Rempe, Ott et al. (2023)	434 HC (6–84)	MEG	aPSD, rPSD	Age, sex	Whole-brain vertex-wise linear and quadratic regressions, hierarchical regression	n/a	Decrease in low-frequency power with age, increase in high-frequency power. Global sex effects on cortical activity	Yes	Yes	No
Wu & Lin (2023)	260 HC (20–70)	raw EEG	aPSD, rPSD	Age, sex	Age-stratification and z-scoring	NeuroGuide DB (Thatcher et al., 1987), MDD (221)	Elevated β and high-β power in the frontal and central lobes.	No	No	Yes
Leroy et al. (2025)	532 HC (8–92)	Raw EEG	rPSD, aperiodic offset exponent	Age, Brain Region	Linear regression	n/a	Age-related decrease in alpha frequency and aperiodic exponent; increase in gamma frequency	Yes	No	No
Tabbal et al. (2025)	499 HC (40–92)	Raw EEG	rPSD, AEC	Age, sex	GAMLSS	Parkinson’s (237, >40) Alzheimer’s (197, >40)	Around 30% of patients exhibited spectral deviations, ~80% showed functional source connectivity deviations. Patient-specific deviations correlated with clinical measures	Yes	Yes	Yes
Ebadi et al. (2025)	448 HC (5–18)	Raw EEG	rPSD, functional connectivity	Age, sex	GAMLSS	ADHD (650), ASD (576), Anxiety (216), HC (112)	Deviations from population norms among patients were highly heterogeneous and frequency dependent. Patient-specific markers demonstrated a correlation with clinical assessments	Yes	Yes	Yes
Q. Dong et al. (2025)	1212 HC (17–30)	Raw EEG	PSI	Age, sex, site (ComBat)	GAMLSS	n/a	Theta (C3–CP2) values decrease with age, beta (C3–CP5) values increase with age. EEG features show specific patterns in attention tasks	No	Yes	No
Woodhouse et al. (2025)	Non-epileptic areas of epilepsy patients, 500 (7–84)	iEEG	log(rPSD)	Age, sex, site	LMM	n/a	The age effect varied by frequency band, but no spatial patterns were observed at the region-specific level	No	Yes	No
Zamanzadeh et al. (2026)	1846 HC (6–88)	MEG	log(PSD), rPSD	Age, sex, site, protocol	Hierarchical Bayesian regression	Parkinson’s disease (160)	Multi-site normative curves. Enhanced detection of deviation. Regional, frequency-specific findings	Yes	Yes	Yes

Criteria include (i) carefully selected reference cohort (HC: healthy control), (ii) appropriate modeling algorithm, (iii) out-of-sample validation of deviation scores. Features: aPSD (absolute power spectral density), rPSD (relative PSD), AER (averaged evoked response), ERP (event-related potential), AEC (amplitude envelope correlation), PSI (phase synchronization index), SES (socioeconomic status). Algorithms: GLM (generalized linear model), GAM (generalized additive model), GAMLSS (Generalized Additive Models for Location, Scale and Shape), LMM (linear mixed model).

In summary, we extensively reviewed 180 studies related to resting-state EEG and MEG-based normative modeling. Of these, 25 satisfied all inclusion criteria. We included only peer-reviewed studies that presented normative models based on carefully selected reference cohorts. Other studies were either cited for their relevance or excluded for lack of alignment with the goal of this review to provide a comprehensive overview of early, contemporary, and future directions in normative modeling of neurophysiological signals.

## 5 Early Studies of Normative Modeling

One of the earliest studies in normative modeling dates back to Matoušek and Petersén (1973), which is the first formal attempt to estimate developmental norms based on EEG signals. Using a cohort of 560 neurotypical individuals aged 1–21 years, they extracted 20 spectral EEG features across various conditions (resting state, sleep, photic stimulation) and applied multivariate analysis to compute age-dependent quotients. Validation was performed on a pathological cohort of 46 patients aged 14–74 years with verified brain lesions, confirming the clinical relevance of the norms (Thatcher & Lubar, 2009). Subsequently, John et al. introduced Neurometrics (1977) (John & Prichep, 1991; John et al., 1977; Mas et al., 1993), using EEG from 144 healthy individuals (ages 20–35 years) to develop quantitative taxonomies of brain activity, with the innovation of examining averaged evoked response characteristics to stimuli, as well as resting-state conditions. The results were compared with a cohort of 177 patients with various neurological diseases. Further validation arose from a lifespan-based study of 280 individuals that included psychiatric and neurological conditions (John & Prichep, 1991), and another that showed that spectral EEG features could distinguish major depressive disorder from primary degenerative dementia with high discriminative power (Deslandes et al., 2004). In 1980, John et al. (1980) expanded the database cross-culturally, collecting EEG from 630 healthy U.S. and Swedish children (6–16 years) and computing 32 spectral features. Although they explored higher-order polynomials, they ultimately opted for simple linear regression and did not model higher variability during childhood. Their results showed replicability across demographic subgroups and were validated on an external cohort of 140 neurotypical children and young adults (aged 5–21 years) and, subsequently, in 2 other studies. One gathered data from 97 (aged 7–11 years) children (Alvarez et al., 1987). Another (Ahn et al., 1980) included patients (8–11 years) with proven learning impairment or assessed risk of development of neurological conditions as well as healthy neurotypical children from multiple sites and studies. Following this foundational work that defined the use of qEEG in computational neuropsychiatry, researchers began applying regression models to map how qEEG features evolve with age. A study (Gasser, Verleger, et al., 1988) investigating this in children and adolescents (158, 6–17 years) found, contrary to previous and subsequent literature, no sex differences and no pubertal spurt. Norms were obtained through polynomial regression of spectral EEG features. The same study yielded different results when paired with principal component analysis to investigate topographic differences in band power and signal coherence across channels (Gasser, Jennen-Steinmetz, et al., 1988). In contrast, a study from 1990 (Harmony et al., 1990) referred to the aforementioned work but confirmed previous findings of age and sex effects on EEG. The research group derived norms from signal spectra of 118 healthy children (6.4–12.9 years) with respect to sex, age, socioeconomic status (SES), and biological risk factors (e.g., malnutrition in pregnancy, perinatal asphyxia, head trauma, etc.). By including SES as a covariate, they found signs of maturation lag in children with lower SES scores. Deviations were found in children with more severe biological risk factors.

In parallel, Thatcher et al. (1979–1987) (Thatcher, 1998; Thatcher et al., 1987; Thatcher & Lubar, 2009) collected resting-state EEG from approximately 1,350 individuals, 620 of whom were healthy and included as reference cohort. Their paper (Thatcher et al., 2003) introduced a normative modeling approach using Gaussian distribution fitting, age stratification, and z-scoring. Cross-validation confirmed the Gaussian nature of derived z-scores, enabling accurate standardization for individualized analysis. This work laid the foundation for a publicly available database (Thatcher & Lubar, 2009) that has since served as a reference for newer models and techniques. For example, the LORETA (Pascual-Marqui et al., 1994) validation study (Thatcher et al., 2005) assessed its reliability, using data from 106 healthy adults (ages 18–82 years), demonstrating how source reconstruction methods can be integrated into normative frameworks as an additional analytical layer. A further compelling use case for this early paradigm is the development of personalized neurofeedback training protocols, informed by normative qEEG databases, which are based on deviations from healthy age-matched norms (Arns & Lyle, 2011; Budzynski, 2009; Koberda et al., 2012; Thatcher & Lubar, 2009).

Between 1988 and 1990, the Cuban Human Brain Mapping Project (CHBM) (Bosch-Bayard et al., 2020; Szava et al., 1989; Valdés et al., 1992) collected EEG from 600 individuals aged 5–80 years, with 211 healthy individuals. Analyses revealed age-related changes in features such as peak α frequency, and statistical parametric maps were created to visualize deviations. CHBM also supported VARETA development (Bosch-Bayard et al., 2001), enabling voxel-based EEG source spectrum estimation and modeling developmental surfaces as functions of both age and frequency (Szava et al., 1994). The study validated the original Cuban norms with a cohort of 129 individuals who suffered childhood malnutrition (5–11 years) and a matched healthy sample (Bringas Vega et al., 2019). From the same epidemiological study, the research group extracted a subset of developmental trajectories (5–12 years) for 165 neurotypical schoolchildren. The childhood study (Amador et al., 1989) improved previous similar work (Matoušek & Petersén, 1973) by adding fitting parameters in the linear regression analyses as well as including ξα -model’s parameters to explore a different feature space. These models decompose the EEG power spectrum into two orthogonal components that are the aperiodic exponent (ξ) capturing the broadband $1\text{​}/\text{​}\omega^{\beta }$ decay across the full frequency range (Pascual-Marqui et al., 1988), and periodic alpha (8–12 Hz) narrow-band oscillatory activity (Miller et al., 2009). This dual parameterization separates scale-free background spectral characteristics from band-specific oscillatory features, extending traditional spectral analytics.

From 2004 to 2008, a second wave of CHBM (Hernandez-Gonzalez et al., 2011; Valdes-Sosa et al., 2021) produced a multimodal dataset of 282 healthy adults (18–68 years), combining high-density resting-state EEG with MRI. Covariates included age, gender, education, ethnicity, handedness, and weight. Norms were extracted using regression models and z-scoring. CHBM’s value has been confirmed in clinical studies, such as Chiarenza’s work (Chiarenza, 2021) identifying qEEG deviations in adolescents with ADHD. During this same period, normative modeling diversified further. Koenig et al. (2002) analyzed EEG microstates, short-lived scalp topographies, across 496 healthy individuals aged 6–80 years. This new approach to EEG analysis showcases the potential of the normative modeling framework as well as its up until today largely untapped EEG feature space. They found systematic age-related changes in microstate dynamics, offering a new dimension for EEG-based developmental modeling. Meanwhile, the early 2000s also saw renewed interest in developmental neuroscience at the neonatal and fetal phase (Mandelbaum et al., 2000; Paul et al., 2006; Selton et al., 2000; West et al., 2006). This movement represented a return to the framework’s original purpose, modeling normative early childhood growth trajectories, but with new computational precision and neuro-psychiatric motivation. Among these works, one stands out as true normative (Otero et al., 2011). It established regression-based EEG developmental equations from a large, representative cohort of 101 healthy infants (15 days–12 months) and validated the resulting spectral-age functions on an independent sample, thereby providing the first quantitative qEEG norms for the first year of life. Alongside analytical advances, standalone software tools emerged (John et al., 1977; Kaiser, 2007). These solutions quantify individual EEG deviations against a reference model, providing patient reports. They have also been used as external validation datasets in some studies (Lorensen & Dickson, 2003; Wu & Lin, 2023). For example, a recent work (Duric et al., 2025) showed significant differences in theta/beta ratio and frontal theta activity in ADHD patients compared with neurotypical individuals, confirming power decrease that correlated with symptom severity as a function of age. However, the resulting scores are often presented with limited transparency regarding underlying modeling assumptions and computational procedures.

In summary, early normative modeling studies laid the foundation for the field’s modern evolution. Beginning in the 1970s and 1980s, researchers increasingly focused on understanding human brain development through EEG functional analysis. These pioneering efforts examined both resting-state and task-based conditions to establish normative baselines for healthy brain function. Fundamental findings emerged and were subsequently validated by later research. The dependency of the EEG spectrum on age and sex was consistently reported, with early developmental trajectories documented and shared across studies (Harmony et al., 1990; John & Prichep, 1991; John et al., 1977). Researchers pursued diverse methodological approaches; some explored neurofeedback and multimodal frameworks (Thatcher, 1998; Valdes-Sosa et al., 2021), while others investigated different feature spaces (Amador et al., 1989; Pascual-Marqui et al., 1988) to identify common patterns of signal changes across the lifespan. Although constrained by the technological limitations of their era, these early models introduced ideas that advanced the field and were later confirmed by more sophisticated analytical pipelines. Importantly, this body of work underscored the value of large-scale initiatives and multi-center collaborations. Above all, it reinforced the pressing need for a robust framework to advance personalized medicine based on electrophysiological brain signals.

## 6 Contemporary Studies of Normative Modeling

In addition to EEG, modalities such as MEG and iEEG have played an increasingly significant role in advancing normative modeling in contemporary studies. A notable example is the Cam-CAN dataset (Cam-CAN et al., 2014), a versatile multimodal normative database from 656 healthy adults (aged 18–87 years) (Brady et al., 2020; Lawn et al., 2024; Xifra-Porxas et al., 2021). The collected MEG data have been used to study age, sex, education, and socioeconomic effects using univariate general linear models (Itälinna et al., 2023). Later, the MNI Open iEEG Atlas (Frauscher et al., 2018) marked a major step in iEEG-based analyses. Built from 106 epilepsy patients’ non-epileptogenic regions (1,785 channels), the study modeled spectral activity during rest and demonstrated region-specific differences. Bernabei et al. (2022) expanded this work using a random forest classifier to identify epileptogenic zones based on deviation from connectivity patterns, especially in the mesial temporal lobe. Similarly, P. N. Taylor et al. (2022) constructed the RAM iEEG database from 234 epilepsy patients and validated it on a secondary cohort. These analyses could predict surgical outcomes, showing that spared regions were more abnormal in patients with persistent seizures. More recently, a multi-site iEEG initiative (Woodhouse et al., 2025) with a cohort of approximately 500 patients modeled spectral activity of healthy cortical areas with linear mixed models. The algorithm included age, sex, and site effect estimation on intracortical measures and showed that the age effect varies by frequency band, but no spatial patterns were observed at the region-specific level. These results remain descriptive and were not tested against an external cohort. That said, it is important to recognize that these cannot truly be considered normative signals, as they are derived from a diseased brain, albeit from regions that appear healthy within that context. Whether it is even possible to isolate genuinely normative activity under such conditions remains uncertain, given the brain’s highly interconnected nature (Engel et al., 2013). When one region is affected, it is arguably more appropriate to regard the entire network as functionally abnormal.

Recent years have brought significant methodological data-driven advances. The ISB-NormDB (Ko et al., 2021), developed in Korea from 1,289 healthy individuals (ages 4.5–81 years), applied generalized additive models to model nonlinear EEG changes across sex and age. Validation showed Z-score deviation trends aligned with clinical abnormalities in ADHD, mild cognitive impairment, and anxiety. The Harmonized-Multinational qEEG Project (M. Li et al., 2022) (HarMNqEEG) gathered EEG features from 1,564 healthy individuals (ages 1–97 years) across 9 countries (Valdes‐Sosa et al., 2022) (Global Brain Consortium). Using Riemannian geometry and additive mixed-effects kernel regression, the project created harmonized developmental norms adjusting for site and device effects. Validation on cohorts with early-life malnutrition and COVID-19-related brain dysfunction showed improved diagnostic precision and brain-age prediction (Jarne et al., 2023). Turning to MEG-based initiatives, the Lifespan MEG dataset (Rempe et al., 2023) modeled age- and sex-related changes in spectral power using linear and quadratic regression on a reference cohort of 434 healthy participants (ages 6–84 years). Findings showed decreased low-frequency power and increased high-frequency activity with age and global cortical sex effects. Parallel to these, in 2023, Taiwan launched a national EEG normative database (Wu & Lin, 2023), with 260 healthy adults (ages 20–70 years), modeled using Gaussian distributions. Validation on a cohort with 221 individuals with major depressive disorder revealed increased β and high-β power in frontal and central lobes, consistent with pathology-related cortical hyperactivity.

Two pilot studies (Janiukstyte et al., 2023; Pastor & Vega-Zelaya, 2023) adhere largely to contemporary normative modeling principles. One study (Pastor & Vega-Zelaya, 2023) examined the normative structure of resting-state EEG using a bipolar montage in 37 healthy individuals aged 10–74 years. Their analysis pipeline incorporated both spectral features and entropy measures, including Shannon’s spectral entropy, to quantify synchronization and the complexity of brain activity. Similarly, the other study (Janiukstyte et al., 2023) generated maps of brain dynamics from 17 healthy participants using source-localized EEG, correlating these with MEG and iEEG recordings. This work further extended the approach to epilepsy, illustrating the potential of normative EEG maps to aid in lateralizing abnormal regions. While both studies reflect a systematic approach to normative modeling, they are limited by the absence of statistically robust norms and large-scale reference cohorts. Nonetheless, they align with the methodological framework evaluated here.

Moving to more recent contributions, in a recently published study (Leroy et al., 2025), 532 (8–92 years of age) neurotypical individuals were characterized based on age-related changes in periodic and aperiodic neural activity in EEG. They used spectral decomposition and linear regression to derive age-adjusted features. However, they opted not to validate or test their findings on an external cohort. As such, while it offers valuable descriptive insights, it falls short when compared with contemporary normative studies. Furthermore, a recent (Hu, Huang, et al., 2025) pre-print article leverages a multi-site EEG dataset from 1966 individuals, aged 5–97 years, across 14 sites. This work employs generalized additive models for location, scale, and shape (GAMLSS) (Stasinopoulos et al., 2018), and proposes a novel interpretable encoder–decoder framework: the Generative Age-dependent brain Network nORmative Model (GANORM) to establish age-dependent normative trajectories of complete brain networks. Although still a pre-print, this approach exemplifies the continued innovation in modeling algorithms and large-scale data integration and aligns with the principles of contemporary normative modeling in EEG and MEG as presented in this review. Unlike opaque machine-learning methods, GAMLSS distributional regression algorithms provide transparent, fully interpretable parameterization while maintaining superior adaptability, and are, therefore, increasingly favored (Q. Dong et al., 2025; Ebadi et al., 2025; Tabbal et al., 2025). Three further studies, each applying similar pipelines to different clinical questions, were published in close succession. The first one (Tabbal et al., 2025) extracted spectral and functional connectivity features from a large multi-site dataset of adults (n = 499, 40–92 years). The adult-life-span trajectories were tested against Alzheimer’s and Parkinson’s diseases patients and the results showed how clinical assessments are in line with neurological functional results, and that functional connectivity deviations show a consistent effect within pathological group. The second (Ebadi et al., 2025) study, with same dataset construction, evaluated measures from a portion of the reference population with age between 5 and 18 years. This study showed how conditions such as ADHD, ASD, and anxiety manifest with high heterogeneity, and individualized biomarker identification provides more insights into the diagnosis and correlates with the clinical test results. The third study (Q. Dong et al., 2025) showcases GAMLSS suitability to construct an accurate normative modeling framework, going beyond developmental trajectories estimation. In the referred paper, spectral functional connectivity measures (e.g., phase synchronization index (PSI)) were used to predict quantiles from a large multi-site sample (n = 1,212, 17–30 years). Site batch effect was accounted for with ComBat algorithm (Fortin et al., 2017; Johnson et al., 2007) in a step prior to GAMLSS training. They then used the fitted normative model to perform posterior feature selection, linking the most deviant features to individual performance on attention task, connecting them to behavioral outcomes. In a fourth study, the authors built MEGaNorm, a large MEG-based normative framework (Zamanzadeh et al., 2025, 2026). Their models were trained on canonical spectral features extracted from a large, multi-site MEG dataset (n = 1,846; ages 6–88 years). Lifespan trajectories were estimated using hierarchical Bayesian regression, accounting for site as a batch effect and explicitly modeling heteroscedasticity, with variance treated as an age-dependent quantity of interest. This framework enabled the mapping of disease-related abnormalities in a Parkinson’s disease cohort (n = 160) and revealed a continuum of patients within a theta–beta deviation space.

Building on early foundational work, contemporary normative modeling has advanced, driven by unprecedented data scale and diversity. This progress has been enabled by large multi-site datasets with harmonized features including MEG and iEEG signals. Methodologically, the field has evolved from simple linear models to nonlinear regression techniques and complex algorithms capable of capturing subtle, individualized patterns beyond average trends. Modern approaches model not only the central tendency but also higher order moments of the distribution (Fig. 2ii). Advanced implementations leveraged variance patterns as quantitative descriptors of covariate-dependent changes in the reference population. Rigorous validation has become more prevalent, ensuring not only the generalizability of models but also their relevance for addressing specific clinical questions and detecting condition-specific deviations. Nevertheless, there remains considerable room for improvement in terms of methodology, reference cohorts, and clinical validation.

## 7 Methodological Advances and Current Applications

Comorbidity and heterogeneity are highly prevalent in neuropsychiatric disorders (Castro et al., 2022; Fried & Nesse, 2015; Fried et al., 2014; Hesdorffer, 2016; Jacobi et al., 2004; Kessler & Merikangas, 2004; Kessler et al., 2005; C. Li et al., 2023; A. F. Marquand et al., 2016; Segal et al., 2023; Thompson, 1994; Verdi et al., 2021, 2023; Winter et al., 2022; Young et al., 2018), posing a major challenge for traditional case–control approaches that rely on discrete diagnostic categories. Normative modeling addresses this limitation by shifting the analytical focus from between-group comparisons to the quantification of individualized deviations from population expectations (N. J. Brown, 2012; Keizer, 2021; Mathur & Sutton, 2017). Within this framework, individuals are characterized relative to a reference distribution rather than assigned to predefined diagnostic groups, enabling the systematic investigation of biological heterogeneity across clinical populations.

Initially, normative modeling relied on simple parametric approaches applied to small, single-site datasets (Bosch-Bayard et al., 2020; John et al., 1977, 1980; Matoušek & Petersén, 1973; Thatcher, 1998; Thatcher et al., 2003) (Supplementary Fig. 2). These early studies often used linear models and relied on narrowly defined “super healthy” reference samples, limiting their generalizability and clinical utility. While instrumental in introducing the concept of population-based electrophysiological norms of the brain, such approaches were constrained by their statistical assumptions (e.g., linearity, homoscedasticity) and by a narrow set of signal features, typically spectral biomarkers extracted from EEG and MEG (Thatcher & Lubar, 2009). Contemporary normative modeling, in contrast, represents a methodological and conceptual leap forward. It leverages large-scale, often multi-site, datasets (Bayer et al., 2022; Kia et al., 2021; Sejnowski et al., 2014), and employs flexible modeling techniques capable of handling complex, nonlinear relationships (Dahl & Hylleberg, 2004; Jarantow et al., 2023; Liang et al., 2022; Stasinopoulos et al., 2018). These improvements allowed for more precise, individualized assessment (Itälinna et al., 2023; Wu & Lin, 2023) to detect atypicality in complex brain disorders (ENIGMA Clinical High Risk for Psychosis Working Group et al., 2024; A. F. Marquand et al., 2016; Rutherford, Kia, et al., 2022; Wolfers, 2019; Wolfers et al., 2018).

These transitions are exemplified by several large-scale initiatives that have advanced EEG- and MEG-based normative modeling. Notable examples include Cam-CAN (Cam-CAN et al., 2014; J. R. Taylor et al., 2017), ISB-NormDB (Ko et al., 2021), and the HarMNqEEG project (M. Li et al., 2022), which established relatively large and demographically diverse reference datasets integrating electrophysiological recordings with multimodal biomedical data (e.g., EEG, MEG, MRI) and demographic information. Using statistical frameworks such as mixed-effects kernel regression, generalized additive models, and multivariate generalized linear models, these studies characterized developmental trajectories of electrophysiological features while accounting for covariates including age, sex, and other relevant factors. Importantly, these models were evaluated in external pathological cohorts, enabling the derivation of individualized deviation scores.

Recent studies employing such frameworks have confirmed earlier observations of systematic age- and sex-related changes in EEG spectral properties (Q. Dong et al., 2025; Ebadi et al., 2025; Ko et al., 2021; Leroy et al., 2025; M. Li et al., 2022; Tabbal et al., 2025). Intracranial EEG investigations have applied similar approaches to identify regional electrophysiological abnormalities in focal epilepsy (Frauscher et al., 2018; P. N. Taylor et al., 2022; Woodhouse et al., 2025). Parallel efforts in MEG have largely followed the same methodological principles, with greater emphasis on source localization and cortical activation patterns (Rempe et al., 2023; J. R. Taylor et al., 2017). Notably, MEG spectral analyses have revealed developmental trajectories that closely mirror those observed in EEG (Rempe et al., 2023; Zamanzadeh et al., 2026), supporting the robustness of normative approaches across electrophysiological modalities.

A key strength of these refined pipelines lies in their clinical validation. Studies examining neuropsychiatric disorders consistently demonstrate systematic deviations of patient populations from normative electrophysiological trajectories (J. R. Taylor et al., 2017; Wu & Lin, 2023). In neurocognitive decline, emerging evidence suggests that individual deviations from normative trajectories may carry prognostic value (Lawry Aguila et al., 2025; Tabbal et al., 2025). In contrast, research in neurodevelopmental conditions frequently reveals pronounced heterogeneity in deviation patterns (Ebadi et al., 2025), highlighting the limitations of traditional case–control designs and underscoring the need for individualized analytical frameworks capable of capturing complex biological variation. Despite these advances, most applications of normative modeling to EEG and MEG have focused on features derived directly from the recorded signals. While such features capture meaningful statistical variation in electrophysiological activity, they often lack a direct mechanistic interpretation. Consequently, biological inference remains largely constrained to the information content of the modality itself, motivating the development of richer analytical strategies that link electrophysiological features more directly to underlying neural mechanisms.

In summary, contemporary normative modeling efforts have substantially improved the resolution at which developmental and functional brain trajectories can be characterized. By enabling individualized deviation mapping, this framework provides a scalable, interpretable, and clinically meaningful alternative to traditional group-based study designs. However, compared with imaging-based normative modeling studies (Bethlehem et al., 2022; Rutherford, Kia, et al., 2022; Rutherford et al., 2023), electrophysiological normative modeling still lacks extensive replication across independent cohorts. Addressing this gap represents an important opportunity for future research and will be critical for strengthening the evidence base and accelerating clinical translation.

## 8 Limitations of Contemporary Approaches

Despite substantial recent advances, several methodological and conceptual challenges continue to limit the scalability and clinical utility of normative modeling. A major obstacle is the lack of standardization across data acquisition protocols (Dickinson et al., 2024; M. Li et al., 2022), preprocessing pipelines (Bigdely-Shamlo et al., 2015; Delorme, 2023; Khosla et al., 2020), and feature extraction methods (Dickinson et al., 2024; Singh & Krishnan, 2023; Stancin et al., 2021). These inconsistencies hinder cross-study comparability and reduce the generalizability of normative models. In electrophysiological research on brain signals, power spectral density and band-wise average power remain the most commonly used EEG features because of their simplicity and extensive validation (Stancin et al., 2021). However, these measures capture only a limited fraction of the information contained in electrophysiological signals (Singh & Krishnan, 2023). Expanding the feature space to include measures of functional connectivity (Imperatori et al., 2019; Ranasinghe & Mapa, 2024), entropy (Cataldo et al., 2024; Erguzel et al., 2020), and other indicators of nonlinear dynamics (Allegrini et al., 2010; Ruiz-Padial & Ibáñez-Molina, 2018) could substantially improve model sensitivity and facilitate the identification of more specific biomarkers for neurological and psychiatric conditions. Harmonized feature extraction pipelines and standardized data formats will, therefore, be essential for enabling large-scale, multi-site normative modeling.

Another persistent limitation concerns the modeling techniques commonly used to estimate normative trajectories. Many early, and even some contemporary, normative models rely on linear regression (John et al., 1977; Thatcher & Lubar, 2009) or stratified analyses (Bosch-Bayard et al., 2020; Thatcher & Lubar, 2009). Such approaches assume linear relationships, homoscedasticity, and Gaussian distributions of the modeled variables, assumptions that are frequently violated in neurophysiological data (Benkeser et al., 2018; De Boer et al., 2024; F. K. Ho & Cole, 2023). Consequently, these models may fail to capture nonlinear developmental trajectories and the substantial heterogeneity present in brain development and pathology. More flexible approaches, including generalized additive models (Ko et al., 2021), Gaussian process regression (Rutherford, Kia, et al., 2022), and hierarchical Bayesian regression (De Boer et al., 2024), additive mixed-effects kernel regression (M. Li et al., 2022; Zavatone-Veth & Pehlevan, 2024), have proven successful in other neuroimaging modalities and are increasingly being applied to EEG and MEG modeling (Ko et al., 2021; M. Li et al., 2022; J. R. Taylor et al., 2017). These methods enable more accurate estimation of centiles across the lifespan and improve the modeling of complex interactions between biological and technical covariates.

Selection bias in reference cohorts represents another important challenge. Many normative datasets are derived from highly selected “super healthy” participants because of strict inclusion criteria (A. F. Marquand et al., 2016). This phenomenon, often referred to as healthy volunteer bias (Green et al., 2022; Oswald et al., 2013), results in datasets that do not adequately represent the broader population and can, therefore, undermine the generalizability of normative models (Bethlehem et al., 2022; Falk et al., 2013). Ideally, reference cohorts should capture the demographic and biological diversity of the populations in which normative models are ultimately applied (A. Marquand et al., 2024; A. F. Marquand et al., 2016; Rutherford, Fraza, et al., 2022). One practical strategy is to aggregate data from multiple studies while explicitly modeling site- and acquisition-related effects as covariates. Because many studies are designed around specific patient groups, their associated control cohorts are typically screened primarily for the disorder of interest and often rely on self-report for other neuropsychiatric conditions. Explicitly modeling differences in acquisition systems and recording environments can, therefore, help absorb latent confounds introduced by heterogeneous study designs and sampling strategies (Cattaneo et al., 2015; A. Marquand et al., 2024; A. F. Marquand et al., 2016, 2019; Thatcher et al., 1987). In practice, however, constructing fully representative reference samples is often infeasible. Transfer learning approaches provide a practical alternative (Gaiser et al., 2024; Kia et al., 2022) by enabling reference-based models to be adapted to specific clinical populations or recording environments while accounting for systematic differences between datasets.

Beyond methodological considerations, the translation of normative modeling into clinical practice raises important interpretational and ethical challenges. Contemporary diagnostic frameworks, including recent revisions of the Diagnostic and Statistical Manual of Mental Disorders (DSM) (American Psychiatric Association, 2013) and the Research Domain Criteria (RDoC) (T. Insel et al., 2010) framework, increasingly emphasize dimensional and transdiagnostic perspectives on mental disorders. Within this context, deviation scores should not be interpreted as direct indicators of disease. Rather, they represent statistical departures from population expectations that may reflect pathological processes, benign individual variation, unmodeled covariates, or technical artifacts. Distinguishing between these possibilities requires careful clinical interpretation and domain expertise (A. F. Marquand et al., 2019; Rutherford et al., 2024; Wolfers et al., 2018). Furthermore, successful integration into clinical workflows will require prospective validation studies demonstrating clinical utility, as well as ethical frameworks for communicating statistical deviations to patients without implying a necessary underlying pathophysiology (C. Fraza et al., 2025; Rutherford, Kia, et al., 2022).

Despite these challenges, contemporary normative modeling has reached a level of methodological maturity that supports increasingly fine-grained characterization of brain trajectories at the individual level. What it lacks, relative to imaging-based frameworks, is a comparable record of replication across independent cohorts, a gap that defines the most pressing research priority for the field. A concerted shift toward standardized data acquisition, harmonized preprocessing pipelines, open-access datasets, and advanced analytical methods will be essential for advancing in evidence-based personalized medicine and accelerating clinical translation (Keizer, 2021; Mathur & Sutton, 2017; Rozich et al., 2004). Although accumulating evidence supports the relevance of this framework for understanding individual variation in brain function, normative modeling remains largely a research agenda with considerable translational potential. With continued advances in machine learning, the expanding availability of large-scale electrophysiological datasets, and improved harmonization strategies, it is increasingly poised to become a central component of individualized brain health assessment.

## 9 Future Directions

The future of normative modeling lies not only in acquiring larger datasets but also in strengthening methodological rigor, statistical innovation, and collaborative infrastructure (Gorgolewski et al., 2016; Poldrack et al., 2024). By aligning with best practices in neuroimaging and embracing modern machine learning approaches, normative modeling is increasingly positioned to become a transformative tool for advancing precision medicine (Fig. 2iii). Open-source and transparent solutions will be essential for fostering collaboration and ensuring scientific reproducibility, as demonstrated across the neuroimaging community (Fischl, 2012; *OpenNeuro*, n.d.; Poldrack et al., 2024; Valdes‐Sosa et al., 2022; Van Essen et al., 2012, 2013). Platforms that integrate end-to-end workflows, from data acquisition and preprocessing to advanced modeling and validation, will allow researchers worldwide to contribute to and benefit from shared resources. Such infrastructures would democratize access to high-quality data and analytical tools, accelerating discovery and innovation across the field (Markiewicz et al., 2021; Pernet et al., 2019; Valdes‐Sosa et al., 2022).

A central challenge in this context is the harmonization of data processing and analysis pipelines. Preprocessing in electrophysiology is widely recognized as both a necessary step and a complex methodological task, leading to a diversity of effective but often incompatible pipelines and tools (Delorme, 2023; Delorme & Makeig, 2004; Gramfort et al., 2014; Oostenveld et al., 2011; Van Es et al., 2025). Two complementary strategies can help balance harmonization with statistical flexibility. First, variability arising from anatomical factors such as skull thickness or head geometry, as well as technical factors including electrode placement and impedance, can be mitigated through post-acquisition harmonization procedures (M. Li et al., 2022; Prichep, 2005). At the same time, the impact of preprocessing choices on the informational content of the signals must be carefully considered (Delorme, 2023; Hu, Ruan, et al., 2025). Establishing standardized preprocessing pipelines would substantially improve the comparability and validation of results across studies. Second, normative models must distinguish meaningful biological variation from nuisance variation. This can to some extent be achieved through explicit covariate modeling procedures in which confounding factors, such as acquisition systems, are treated as batch or random effects (De Boer et al., 2024; Kia et al., 2022; Rutherford, Kia, et al., 2022). Additional variables including data quality metrics, preprocessing parameters, and experimental paradigms (e.g., eyes-open versus eyes-closed recordings) can also be incorporated during model training to reduce bias introduced by heterogeneous datasets. Combining harmonized preprocessing with explicit modeling of residual site-specific effects helps ensure that normative models capture genuine neurophysiological variation rather than technical artifacts, thereby improving robustness and translational value.

Beyond infrastructure and harmonization, expanding the repertoire of electrophysiological features represents an important direction for future development. Historically, both early and contemporary normative modeling studies have relied predominantly on temporal and spectral characteristics of brain signals, such as quantitative EEG measures. Although Fourier-domain analyses remain fundamental to signal processing and neuroscience (Benwell et al., 2020; Stancin et al., 2021; Tenev et al., 2025), a growing body of literature highlights the value of complementary descriptors. Measures of nonlinear dynamics, entropy, and signal complexity (Cataldo et al., 2024; Erguzel et al., 2020; Frohlich et al., 2022; Ruiz-Padial & Ibáñez-Molina, 2018; Tenev et al., 2025), as well as functional connectivity metrics (Garcia-Cordero et al., 2024; Imperatori et al., 2019), have demonstrated considerable promise in capturing aspects of brain function not reflected in traditional spectral measures (Ebadi et al., 2025; Erguzel et al., 2020; Franzmeier et al., 2017; Frohlich et al., 2022; Garcia-Cordero et al., 2024; Imperatori et al., 2019; Jensen et al., 2025; G. Li et al., 2023). Microstate dynamics provide an additional perspective by describing the temporal organization and switching of global brain states, revealing disruptions in large-scale network dynamics (Andreou et al., 2014; Bagdasarov et al., 2022; Koenig et al., 2002).

Advances in computational techniques further enable the derivation of multidimensional descriptors of brain function that extend beyond conventional feature spaces (Chaudhary, 2025; García-Ponsoda et al., 2023; Islam et al., 2019; Kandel, 2014). These approaches can be applied to both resting-state and task-based recordings, including event-related potentials, and aim to extract higher-level representations of neural signals. Analytical decisions regarding feature extraction and spatial representation critically shape both the behavior of normative models and their interpretability. Each feature class probes distinct dimensions of neural function, and individuals may, therefore, show normative profiles in some domains while displaying abnormalities in others. Modeling multiple feature spaces simultaneously allows a more comprehensive characterization of individual deviations and helps distinguish feature-specific alterations from global dysregulation.

Task-based electrophysiological data offer additional opportunities to enrich normative modeling frameworks. Event-related potentials provide context-sensitive features by isolating stimulus-locked neural responses associated with sensory processing, attention, and cognitive control (De Blasio & Barry, 2013; Harper et al., 2017; Morand-Beaulieu et al., 2022). The increasing availability of large task-based EEG datasets opens new possibilities for modeling brain responses under specific cognitive or behavioral conditions (Grootswagers et al., 2022; Hebart et al., 2023; Sabo et al., 2025; Taylor et al., 2017). These datasets allow the identification of context-dependent neural signatures and may increase sensitivity in detecting deviations from normative patterns. Moreover, the growth of large-scale task datasets has made it feasible to train deep neural networks capable of capturing complex patterns in electrophysiological data (Banville et al., 2025). Such models are particularly useful for representing inter-individual variability in responses to stimuli or task demands that may not be fully observable in resting-state recordings. Nevertheless, variability in experimental design, preprocessing procedures, and event annotation continues to hinder the development of fully automated and generalizable pipelines (Poldrack et al., 2011; Turner & Laird, 2012). One possible workaround involves the use of standardized paradigms such as the oddball paradigm (Carbajal & Malmierca, 2020; Kutas et al., 2012), which reliably elicits characteristic EEG responses largely independent of specific task implementations (Kamp et al., 2023; Morand-Beaulieu et al., 2022). Integrating task-based and resting-state recordings may, therefore, substantially enhance the resolution of normative models by capturing a broader spectrum of brain dynamics.

Alongside advances in feature extraction, progress in statistical modeling and machine learning is expanding the methodological foundations of normative modeling. Traditional approaches often rely on manually specified priors and relatively simple parametric models. Increasingly, more flexible analytical techniques are being explored to better capture inter-individual variability in complex neurological data (Z. Dong et al., 2023; Malkiel et al., 2022). Non-parametric approaches such as locally weighted regression (Jacoby, 2000) allow smooth trajectories to be fitted without assuming predefined functional forms and can, therefore, be particularly suitable for normative modeling (Jiang et al., 2024; Van der Elst et al., 2013). At the same time, recent developments in generative modeling provide powerful alternatives for learning complex data distributions (Frotscher et al., 2023; Schmidl et al., 2022). Conditional variational autoencoders (Kia & Marquand, 2018b; Sampaio et al., 2024; You et al., 2025), for example, can learn conditional representations of electrophysiological data and generate representative samples reflecting population variability. Similarly, score-based diffusion models (J. Ho et al., 2020; L. Yang et al., 2024) and flow-matching approaches (J. Ho et al., 2019; Lipman et al., 2023) have demonstrated considerable potential for modeling high-dimensional data distributions while preserving uncertainty estimates that are essential for normative inference.

Extending normative modeling toward multivariate and high-dimensional frameworks represents another promising direction. Deep learning-based approaches have already shown success in structural and functional neuroimaging (Kia & Marquand, 2018b; Kumar et al., 2023; Pinaya et al., 2019; Sampaio et al., 2024; You et al., 2025) by learning hierarchical representations that capture complex nonlinear relationships between brain features and clinical phenotypes. In such models, biological features are embedded in lower-dimensional latent spaces that preserve meaningful variation while enabling individual-level deviation modeling (Kia & Marquand, 2018a; Kumar et al., 2023; Zabihi et al., 2021, 2025). These frameworks can explicitly account for structured spatial dependencies across brain regions and integrate information across modalities, enabling joint modeling of structural–functional coupling (Fotiadis et al., 2024; Zhang et al., 2024). Despite the inherent advantages of electrophysiological data for capturing neural dynamics, comparable multivariate normative modeling frameworks remain relatively underexplored in EEG and MEG research (Dursun et al., 2025), although related analytical strategies are widely used in other domains (Saeidi et al., 2021).

These developments shift the focus from simple feature extraction toward feature engineering and representation learning. Rather than treating measures as isolated biological descriptors, features are organized and combined according to their statistical relationships. In this context, direct biological interpretation of individual features may be partially replaced by the interpretation of latent representations within lower-dimensional spaces. Maintaining interpretability, understood here as the real-world translational utility of model outputs, remains a key challenge (Chen et al., 2024). Successfully addressing the latter holds substantial promise for capturing the multidimensional complexity of electrophysiological phenotypes while preserving clinical relevance.

Another important frontier concerns the transition from cross-sectional to longitudinal normative modeling. Most current normative models are based on cross-sectional data, which has proven valuable for characterizing population variability but remains limited in its ability to capture developmental and time-varying processes. Increasingly, researchers are exploring strategies that integrate cross-sectional normative models with longitudinal observations to improve predictive accuracy over time. For example, models trained on cross-sectional datasets can be used to generate expected developmental trajectories that are subsequently evaluated within longitudinal study designs (Rehák Bučková et al., 2025). Other approaches incorporate baseline measurements directly into normative estimates (Alden et al., 2022) or map disease progression by projecting individual trajectories onto normative reference spaces (Berthet et al., 2024; Cirstian et al., 2026; Kim et al., 2025; Pinaya et al., 2021). Berthet et al. (2024) combined longitudinal neuroimaging data with normative modeling and identified distributed cortical deviations in schizophrenia that attenuated over time alongside symptom improvement, challenging strictly neurodegenerative accounts of the disorder. Similarly, Lawry Aguila et al. (2025) constructed multimodal normative reference models in Alzheimer’s disease that revealed regionally specific and progressively evolving deviations in at-risk individuals, advancing early detection and supporting preclinical intervention strategies.

More broadly, there is ongoing debate regarding whether cross-sectional norms alone can adequately capture dynamic developmental processes, particularly during periods of rapid brain change such as adolescence or aging (Korbmacher et al., 2025; Y. Yang et al., 2026). Evidence increasingly suggests that integrating cross-sectional and longitudinal approaches may provide a more nuanced and accurate representation of brain development and disease trajectories. Although most work in this area has focused on MRI, the same principles could be extended to electrophysiology, representing an important avenue for future research.

A further challenge involves capturing the temporal structure of brain activity. One promising direction is to model neural signals as dynamical systems (Brenner et al., 2024; Durstewitz et al., 2023; Y. Yang et al., 2026; Y. Yin et al., 2021). Within this framework, brain dynamics describe the evolving patterns and transitions of neural activity as the brain responds to internal states and external stimuli over time. One strategy involves discretizing continuous time series into recurring brain states such as metastable states or attractors (R. Brown, 2006; Finn, 2017; Tang et al., 2012; Y. Yang et al., 2026). Mapping continuous dynamics onto discrete states enables the characterization of normative patterns of state occurrence, transitions, and dwell times, deviations from which may indicate atypical development or pathology. Such metastable dynamics have been observed across multiple modalities including EEG, MEG, and fMRI (Allegrini et al., 2010; Bapat et al., 2024; Deco et al., 2017; Fingelkurts & Fingelkurts, 2017; Freeman & Holmes, 2005; Tang et al., 2012; Wens et al., 2019; Werner, 2007; Y. Yang et al., 2026) and are often characterized by recurring patterns of functional connectivity, phase synchrony, or latent neural manifolds. Methods such as Hidden Markov Models, Leading Eigenvector Dynamics Analysis (LEiDA), and clustering of dynamic connectivity patterns are widely used to identify these transient states (Alonso Martínez et al., 2020; Baker et al., 2014; Kringelbach & Deco, 2020; Roberts et al., 2019; Vidaurre et al., 2017; Woolrich et al., 2004). Metrics including fractional occupancy, transition probabilities, and entropy of state sequences provide insight into the stability and richness of an individual’s brain dynamics and have been associated with aging, psychiatric disorders, and neurodevelopmental conditions such as autism spectrum disorder and ADHD (Pei et al., 2023; Sase & Kitajo, 2019, 2021). A complementary direction focuses on predicting the temporal evolution of neural signals. Predictive normative models aim to learn the underlying dynamics governing brain activity in order to detect deviations from expected trajectories. Models such as LEADS and CODA (Brenner et al., 2024; Kirchmeyer et al., 2022; Y. Yin et al., 2021) have been proposed to predict individual biological time series while capturing both short-term fluctuations and longer-term trends. Situating deviations within their temporal context enhances sensitivity to subtle disruptions in neural dynamics and improves interpretability. In a recent study by Yang et al. (2026), normative modeling of brain dynamics across the lifespan was used to derive individualized reference trajectories from resting-state fMRI, enabling the quantification of individual-level deviations from typical patterns. The framework was trained on a large-scale cohort and rigorously evaluated on independent, out-of-distribution datasets acquired at previously unseen sites and scanners, demonstrating strong robustness and generalizability. Such approaches hold considerable translational potential for the early detection of atypical brain changes and for longitudinal monitoring of disease progression or treatment effects in clinical populations. Moreover, this work provides a conceptual foundation for extending similar frameworks to MEG and EEG, which could enable more scalable and cost-effective monitoring of brain network dynamics in response to treatment.

Having said that, translating normative modeling into routine clinical practice remains an important, but still challenging, goal. Although EEG-based normative modeling has advanced considerably, its direct application in guiding treatment strategies remains limited. One potential avenue involves neurofeedback interventions, where deviations from normative electrophysiological patterns could guide personalized training protocols aimed at restoring typical activity patterns. More broadly, normative modeling provides stratification markers that quantify how an individual’s neurobiological profile relates to population variability (C. Fraza et al., 2025; A. F. Marquand et al., 2019; Rutherford et al., 2023). These deviation scores are continuous, interpretable, and contextualized, capturing both pathological and non-pathological variation. However, the personalized nature of such approaches presents challenges for validation using traditional group-level clinical trial designs (Hamburg & Collins, 2010). As a result, translating normative modeling into routine clinical practice will likely require new evaluation frameworks capable of assessing individualized treatment responses.

Taken together, normative modeling has evolved into a powerful framework for characterizing individual variability in brain structure and function. Its continued development will depend on advances in data harmonization, feature representation, statistical modeling, and longitudinal analysis. Importantly, future clinical applications should position normative models as tools for interpretation and decision support rather than definitive diagnostic instruments, aligning with the growing recognition that individual-level heterogeneity cannot be adequately captured by traditional group-based analyses alone.

## 10 Conclusion

Contemporary normative modeling emphasizes the transition from classical statistical approaches based on group averages toward personalized estimates. While this transformation is somewhat established in neuroimaging, neurophysiological signals have yet to fully embrace it. In this work, we introduce a definition of contemporary normative modeling and chart its progression, from early foundational studies to emerging trends, with a specific focus on EEG- and MEG-based modeling frameworks. The integration of big data and machine learning marks only a fraction of the progress required. We advocate for greater standardization and harmonization in data acquisition and storage practices, expanded exploration of diverse feature domains, and the adoption of advanced methodologies. Taken together, these efforts position normative modeling of neurophysiological signals as a critical and cost-effective enabler of precision medicine in routine clinical practice, due to its ability to parse out differences at the level of the individual.

## Supplementary Material

Supplementary Material

## Data Availability

This review used no new or pre-existing data beyond the cited manuscripts and their associated dataset information, all referenced accordingly. No code was developed for or is fundamental to the reproducibility of this study.
