# Supplementary material for: From early to contemporary normative modeling: Mapping individual differences in neurophysiological signals
Source: Imaging Neurosci (Camb). 2026 Jun 15;4:IMAG.a.1269. doi: 10.1162/IMAG.a.1269 (PMC13271153; doi:10.1162/IMAG.a.1269)
Supplement: Supplementary Material [file IMAG.a.1269_supp.pdf]

Supplementary Materials for "***From Early to Contemporary Normative Modeling: Mapping Individual Differences in Neurophysiological Signals***" by Francesco Antonio Mallus et al.

Summary of Supplementary Materials: The supplementary materials provide a structured overview of the state of electrophysiological normative modeling. Supplementary Figure 1 presents the systematic selection and review process for EEG, iEEG, and MEG studies, including details of the literature search, screening, and inclusion criteria. Studies are organized according to three central components of contemporary normative modeling: database and cohort selection, modeling approach, and validation procedures. This review highlights the pioneering contributions of early work while also identifying substantial methodological gaps relative to current standards. Supplementary Figure 2 summarizes contemporary normative modeling efforts across multiple dimensions. Together, these supplementary materials provide both a historical perspective on the development of electrophysiological normative modeling and a critical appraisal of current practices, motivating the need for harmonized, large-scale, and computationally advanced approaches.

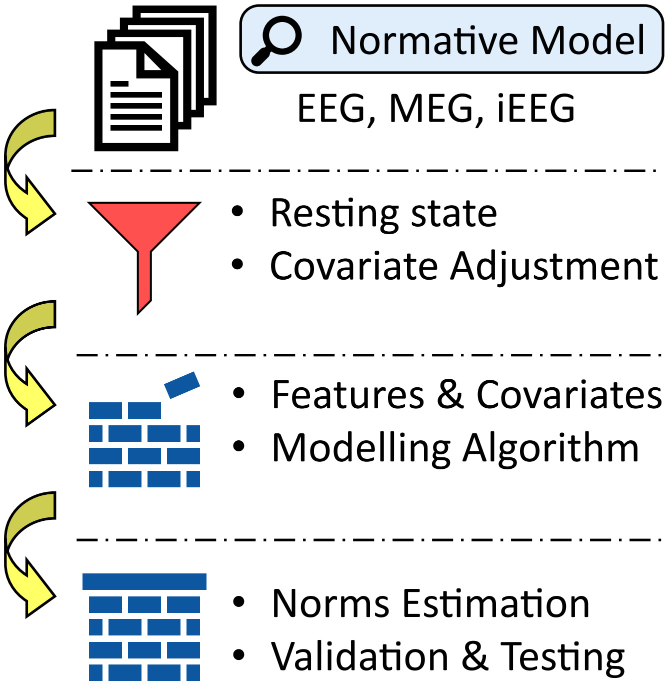

Supplementary Figure 1: Visual summary of the systematic selection and review process for electrophysiological normative modeling studies (EEG, iEEG, and MEG). The literature search began with a broad set of publications identified through major databases (PubMed, Web of Science, Scopus) using terms related to normative modeling and electrophysiology. The initial pool of studies was screened for basic relevance, focusing on resting state and task related EEG, intracranial EEG (iEEG), and MEG. After removing duplicates, reviews, and unrelated articles, the remaining studies were filtered according to specific inclusion criteria, including the use of electrophysiological data in healthy reference samples or patient cohorts, attempts to establish or apply normative models, and the reporting of reference cohort characteristics, modeling approaches, and validation procedures. The final set of eligible studies was then systematically organized around the three core components of contemporary normative modeling: database and cohort selection, including sample size, representativeness, and demographic balance; norm creation and modeling approach, referring to statistical or machine learning frameworks used to derive conditional population norms; and validation procedures, including internal checks, cross validation, and out of sample generalization of deviation scores. This structured review highlights both the pioneering role of early electrophysiological normative modeling and the substantial gaps in methodological rigor relative to current standards, thereby motivating the need for large scale, harmonized, and computationally advanced approaches in the present project.

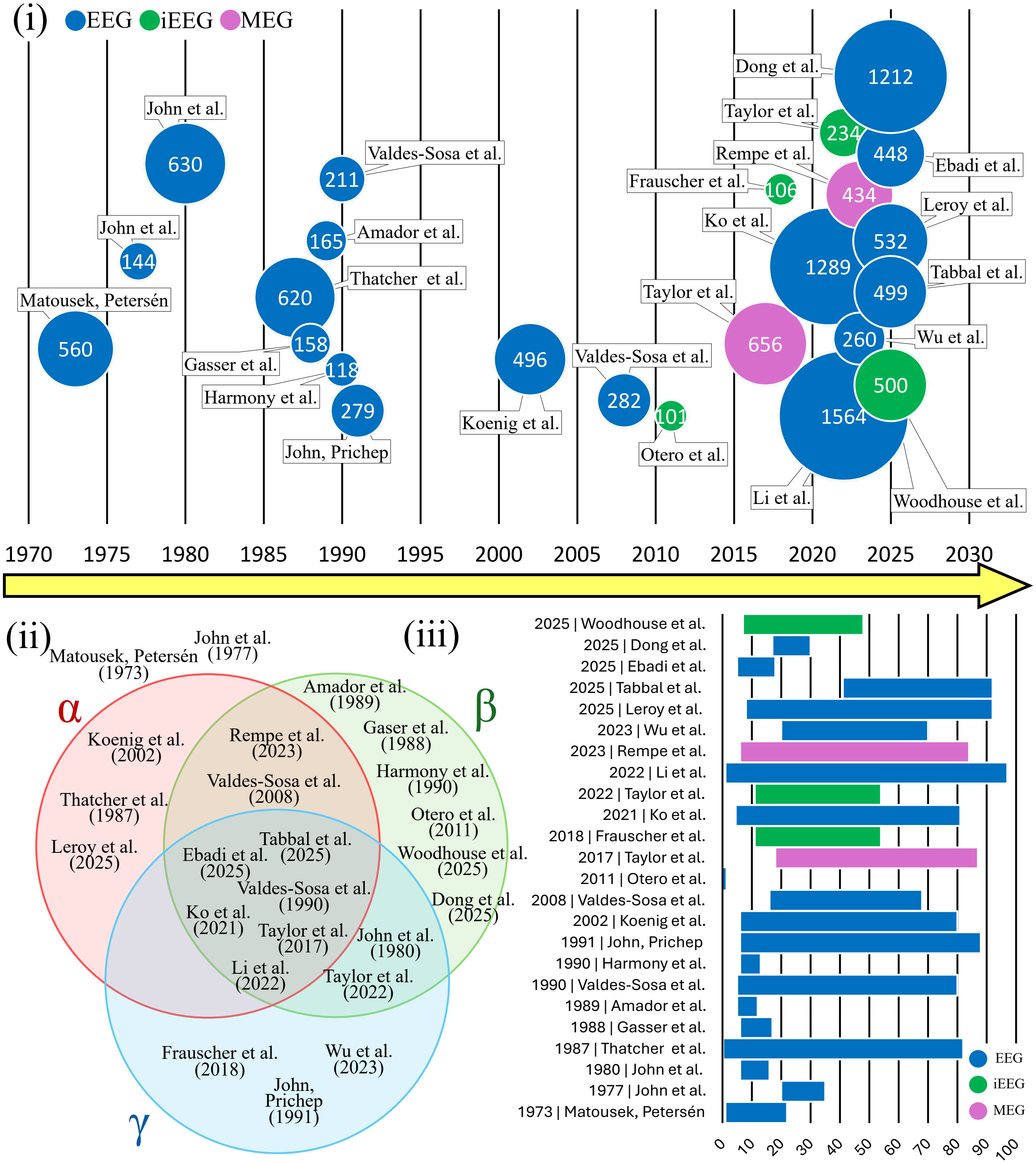

Supplementary Figure 2: Overview of normative modeling in electrophysiology. (i) Timeline and cohort size of normative EEG and MEG databases. Each database is represented as a bubble positioned along the timeline, with bubble size proportional to the reported reference cohort size. **(ii)** Adherence to contemporary normative modeling standards. A Venn diagram illustrates which of the three key criteria are fulfilled by each study. Criteria: α = carefully selected reference cohort; β = appropriate modeling algorithm; γ = out-of-sample validation of deviation scores. Only three studies satisfy all three criteria, whereas the remaining studies fall short on one or more dimensions. **(iii)** Reference cohort age ranges. An overview of the age ranges covered by each study, highlighting a recent shift toward larger and more age-diverse cohorts.
